# Trends of risk behaviors in adolescents: a 10-year study in a representative sample of Tuscany, Italy

**DOI:** 10.1093/eurpub/ckac131.459

**Published:** 2022-10-25

**Authors:** V Lastrucci, M Lazzeretti, F Innocenti, A Berti, C Silvestri, A Schirripa, S Paoli, C Lorini, F Voller, G Bonaccorsi

**Affiliations:** 1 Epidemiology Unit, Meyer Children’s Hospital, Firenze, Italy; 2 Epidemologic Observatory, Regional Health Agency of Tuscany, Firenze, Italy; 3 Medical Specialization School of Hygiene and Preventive Medicine, University of Florence, Firenze, Italy; 4 Department of Health Sciences, University of Florence, Firenze, Italy

## Abstract

**Background:**

The aim of the study was to evaluate the trends of prevalence of several health risk behaviors (HRBs) and health conditions over a 10-years period in a representative sample of adolescents of Tuscany Region, Italy.

**Methods:**

The study had a repeated cross-sectional design, data from the last four survey waves of EDIT surveillance (2008-2018) were used. EDIT surveillance investigates HRBs in a representative sample of students attending the upper secondary schools of Tuscany. Prevalence of 17 HRBs and health conditions were considered and analyzed by age, sex, and socioeconomic status (SES).

**Results:**

A total of 21.943 students were surveyed from 2008 to 2018. Declining trends in the participation in smoking, cocaine use, driving under the influence of alcohol and drugs, and problem gambling were observed, while alcohol abuse and at-risk sexual behaviors remained unchanged or increased during the study period. During the most recent survey males resulted more frequently involved in most of the HRBs, while females more frequently reported physical inactivity, regular smoking and not using a condom. Female participation in smoking and alcohol abuse behaviors, fruit and vegetables consumption, and bullying worsened over the study period. Smoking, poor dietary habits, physical inactivity, high distress level, and obesity were more frequently observed in low SES students than in high SES students.

**Conclusions:**

In conclusion findings showed various different tendencies in adolescent participation in HRBs over the course of the last decade; concerning trends in at-risk sexual behaviors and alcohol consumption and females’ risk-taking behavior on the rise require careful monitoring and intervention.

**Key messages:**

• Distinct tendencies according to sex, socio-economic condition and specific health risk behavior were observed in adolescent participation in health risk behaviors over the course of the last decade.

• Health promotion and prevention interventions tailored on specific health risk behaviors and population groups are needed.

